# Analysis of Processing, Post-Maturation, and By-Products of shRNA in Gene and Cell Therapy Applications

**DOI:** 10.3390/mps8020038

**Published:** 2025-04-07

**Authors:** Zhenyi Hong, Nikola Tesic, Xavier Bofill-De Ros

**Affiliations:** 1 Department of Molecular Biology and Genetics, Aarhus University, 8000 Aarhus, Denmark; 2 Seven Bridges Genomics Inc., Cambridge, MA 02138, USA

**Keywords:** shRNA, miRNA-based shRNAs, processing, post maturation, RNA extraction, library preparation, bioinformatic analysis

## Abstract

Short hairpin RNAs (shRNAs) are potent tools for gene silencing, offering therapeutic potential for gene and cell therapy applications. However, their efficacy and safety depend on precise processing by the RNA interference machinery and the generation of minimal by-products. In this protocol, we describe how to systematically analyze the processing of therapeutic small RNAs by DROSHA and DICER1 and their incorporation into functional AGO complexes. Using standard small RNA sequencing and tailored bioinformatic analysis (QuagmiR), we evaluate the different steps of shRNA maturation that influence processing efficiency and specificity. We provide guidelines for troubleshooting common design pitfalls and off-target effects in transcriptome-wide profiling to identify unintended mRNA targeting via the miRNA-like effect. We provide examples of the bioinformatic analysis that can be performed to characterize therapeutic shRNA. Finally, we provide guidelines for troubleshooting shRNA designs that result in suboptimal processing or undesired off-target effects. This protocol underscores the importance of rational shRNA design to enhance specificity and reduce biogenesis by-products that can lead to off-target effects, providing a framework for optimizing the use of small RNAs in gene and cell therapies.

## 1. Introduction

Gene therapy vectors are designed to deliver genetic material such as DNA or RNA into a patient’s cells to replace, repair, or modulate defective genes, ultimately achieving therapeutic outcomes [1]. These therapies fall into two main categories: nucleic acid-based approaches and genetically engineered cell therapies [2]. Within this landscape, short hairpin RNAs (shRNAs) have emerged as potent tools for gene regulation through the RNA interference (RNAi) pathway [3]. By harnessing the ability of shRNAs to target and degrade specific RNA transcripts, these molecules enable the precise modulation of gene expression. This precision is critical for addressing diseases characterized by aberrant gene activity and holds significant promise for the development of advanced cell therapies. Notable therapeutic applications include cancer, genetic disorders, and viral infections, with shRNAs demonstrating particular utility in immune-oncology targets such as lysine-specific demethylase 1 (LSD1) [4], programmed death-1 (PD-1) [5], and a range of antiviral targets [6].

The design of shRNAs and subsequent processing by the cellular RNA interference machinery is a crucial determinant of their silencing efficiency and safety (Table 1). Optimal outcomes require not only effective target knockdown but also the minimization of off-target effects and cellular toxicity. Achieving this balance necessitates the careful design and thorough evaluation of shRNA constructs to ensure both potency and specificity. shRNA maturation into active small interfering RNAs (siRNAs) relies on precise processing by the microRNA biogenesis pathway [7]. This process involves the critical interplay between shRNAs and key microRNA processing enzymes such as DROSHA and DICER1. This interaction becomes particularly significant in the context of gene therapy vectors, especially those employing complex designs, such as microRNA-based scaffolds or constructs encoding multiple hairpins [8].

**Figure 1:**
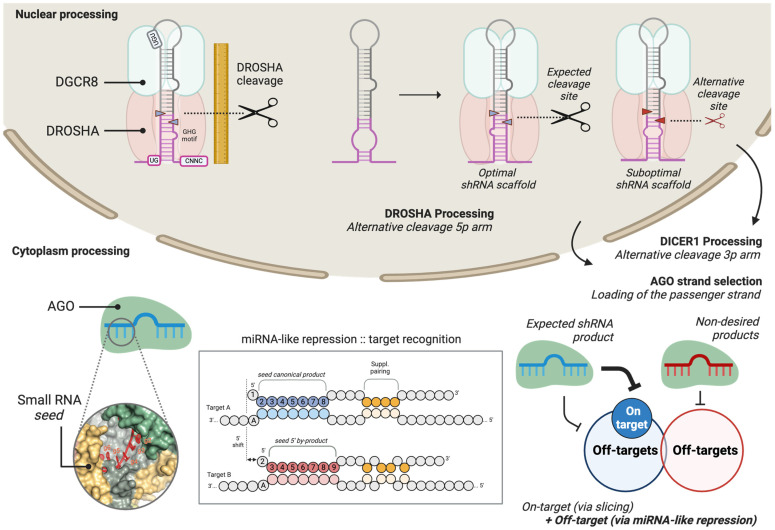
Cellular processing of shRNAs and sources of by-products; alternative processing of shRNAs and its impact on target specificity. During nuclear processing, DROSHA and DGCR8 cleave the pri-shRNA at the expected site in an optimal scaffold, whereas suboptimal scaffolds may lead to alternative cleavage on the 5p arm, generating unintended by-products. In the cytoplasm, DICER1 further processes the shRNA, where alternative cleavage on the 3p arm or passenger strand loading into AGO can lead to non-desired products. These misprocessed shRNA fragments can recognize unintended RNAs via miRNA-like repression, expanding the target repertoire and contributing to off-target effects. Proper shRNA design is critical to minimize these unintended outcomes.

Briefly, shRNA designs can be embedded in longer primary transcripts (pri-shRNAs) that undergo cellular capping and polyadenylation, requiring processing by the DROSHA–DGCR8 complex [3,9]. DROSHA cleaves these primary transcripts, excising one or multiple shRNAs from clusters of hairpins [8]. Proper processing of pri-shRNAs is facilitated by several regulatory steps, including the binding of cofactors to the DROSHA–DGCR8 complex, which enhances the efficiency and specificity of cleavage [10,11]. Both the sequence context of the pri-shRNA and interactions with cofactors play critical roles in determining the dynamics and accuracy of this processing [12] (Figure 1).

Following nuclear processing, the excised shRNA is transported to the cytoplasm by Exportin-5, where it undergoes further maturation by DICER1 into a double-stranded duplex of 21–25 nucleotides. The precise recognition and cleavage by DICER1 depend on specific structural features, such as the 2-nucleotide overhangs left by DROSHA (or transcription termination in Pol III-driven shRNAs), as well as the presence of a 5′ phosphate group and a 3′ hydroxyl group. Structural studies of DICER1 and its hairpin substrates have revealed conformational changes that are necessary to achieve an active dicing state [13,14,15,16]. During this process, specific domains within DICER1 stabilize the shRNA substrate in an optimal conformation for cleavage influenced by structural features and sequence motifs such as the GYM motif [17,18].

This precise cleavage generates a duplex that is subsequently loaded into Argonaute proteins (AGO1–4) to form the RNA-induced silencing complex (RISC). However, errors in shRNA processing can lead to significant issues, such as the production of siRNAs with altered target specificities (“seed shift”) or changes in the strand incorporated into AGO proteins (“arm switch”). These misprocessing events can result in unintended off-target effects, highlighting the importance of optimizing shRNA design and biogenesis [19].

This protocol outlines a systematic approach for evaluating shRNA processing and functionality within gene and cell therapy applications. By leveraging small RNA sequencing and tailored bioinformatic analysis using QuagmiR [20], we assess the key parameters of shRNA biogenesis, including processing efficiency, incorporation into the RNA-induced silencing complex (RISC), and its downstream effects on gene expression.

## 2. Experimental Design

**Figure 2:**
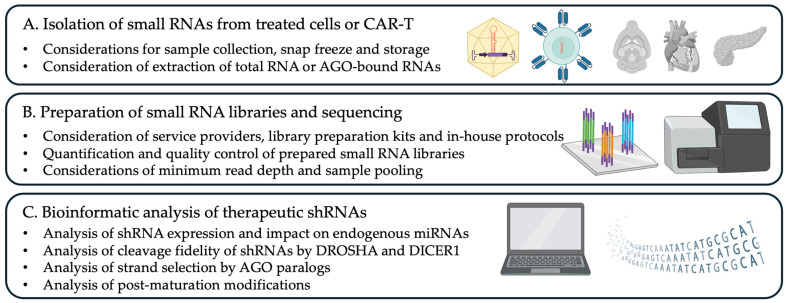
Schematic representation of the workflow for the analysis of candidate shRNAs used in gene and cell therapy applications. The pipeline outline in this protocol contains considerations regarding the sample preparation, construction of small RNA libraries, and computational analysis using established cloud-based open software and custom scripts.

To generate a small RNA library, total RNA is first extracted from biological samples (e.g., shRNA constructs and mock treatments) using methods optimized for small RNA preservation (Section 3.1). Adapters are then ligated to the 5′ and 3′ ends of the small RNA molecules using RNA ligases, leveraging their chemically compatible termini (Section 3.2.1 and Section 3.2.2). The resulting RNA-adapter constructs undergo reverse transcription (Section 3.2.3) followed by PCR amplification with a minimal number of cycles to reduce sequence bias while generating sufficient material for downstream purification (Section 3.2.4). This protocol also includes detailed instructions on how to perform specific data analysis tailored to the study of the biogenesis of shRNAs and its potential impact on endogenous miRNA production (Figure 2).

Accurate shRNA quantification relies on minimizing the biases introduced during library preparation, including adapter ligation, PCR amplification, and sequencing depth variability, which can affect both expression measurements and off-target predictions. Ligation biases arise from the sequence-dependent preferences of RNA ligases, potentially leading to underrepresentation of certain shRNA species. To mitigate this, optimized adapter chemistries or randomized adapter sequences can be used to ensure a more uniform capture of small RNA species [21]. PCR amplification biases may further distort quantification by preferentially amplifying certain sequences, particularly those with high GC content or secondary structures. The incorporation of unique molecular identifiers (UMIs) in the initial adapters helps correct for these biases by distinguishing true biological sequences from PCR duplicates. Additionally, sequencing depth variability can impact the detection of low-abundance shRNA by-products and confound off-target analyses. To address this, maintaining consistent library input amounts, randomizing sample processing order, and applying computational batch correction methods are recommended. Quality control measures such as gel purification (Section 3.2.5) and bioanalyzer-based assessment further enhance the uniformity and reliability of small RNA libraries. Finally, synthetic spike-ins should be carefully selected to match the sequence characteristics of endogenous small RNAs, improving normalization and reproducibility across experiments [22].

### 2.1. Materials

1. TRIzol Reagent (Thermo Fisher Scientific, Waltham, MA, USA; Cat. no.: 15596026)

**ALTERNATIVE:**

miRNeasy Kit for miRNA Purification (Qiagen, Hilden, Germany, Cat. no.: 217084)

2. QIAseq miRNA Library Kit (Qiagen, Germany, Cat. no.: 331502)

**ALTERNATIVES:**

TruSeq Small RNA Library Prep Kit (Illumina, San Diego, CA, USA, Cat. no.: RS-200-0036)

NEBNext Small RNA Library Prep (NEB, Ipswich, MA, USA, Cat. no.: E7330S)

3. RNA 5′ Adapter (RA5) (7.5 μM)

/5AmMC6/rCrUrArCrArCrGrArCrGrCrUrCrUrUrCrCrGrArUrC*rU

Note that there is a phosphorothioate bond between the last C and U.

4. RNA 3′ Adapter (RA3) (5 μM)

/5rApp/CTGTTAACN15TGGAATTCTCGGGTGCCAAGGC/3ddC/

5. RT Primer

CCTTGGCACCCGAGAATTCCA

6. PCR5 Primer

AATGATACGGCGACCACCGAGATCTACACTCTTTCCCTACACGACGCTCTTCCGATCT

7. Stop solution (STP) (15 μM)

rGrUrUrArArCrArGrCrCrArCrGrUrUrCrCrCrGrUrGrG

8. PCR3 Primer

CAAGCAGAAGACGGCATACGAGATNNNNNNGTGACTGGAGTTCCTTGGCACCCGAGAATTCCA

Note that N stands for Illumina sequencing indices

(e.g., Index 1: ATCACG; Index 2: CGATGT)

9. 10× T4 RNA Ligase 1 buffer (NEB, Ipswich, MA, USA, Cat. no.: M0204L)
10. RNase Inhibitor (NEB, Ipswich, MA, USA, Cat. no.: M0314L)
11. T4 RNA Ligase 2, Deletion Mutant (NEB, Ipswich, MA, USA, Cat. no.: M0242S)
12. Adenosine 5′-Triphosphate (ATP) (NEB, Ipswich, MA, USA, Cat. no.: P0756S)
13. T4 RNA Ligase (NEB, Ipswich, MA, USA, Cat. no.: M0437M)
14. SuperScript IV Reverse Transcriptase (Thermo Fisher Scientific, Waltham, MA, USA, Cat. no.: 18091050)
15. Phusion High-Fidelity DNA Polymerase (NEB, Ipswich, MA, USA, Cat. no.: M0530L)
16. RNAlater (Thermo Fisher Scientific, Waltham, MA, USA, Cat. no.: AM7020)
17. Blue Juice Loading Buffer (10×) (Thermo Fisher, Waltham, MA, USA, Cat. no.: 10816015)

### 2.2. Equipment

- NanoDrop (Thermo Fisher Scientific, Waltham, MA, USA; Cat. no.: ND-ONE-W)
- Bioanalyzer (Agilent, Santa Clara, CA, USA; Cat. no.: G2939BA)
- Safe Imager 2.0 (Thermo Fisher Scientific, Waltham, MA, USA; Cat. no.: G6600EU)
- ChemiDoc (BioRad, Hercules, CA, USA; Cat. no.: OI91XQ15)

## 3. Procedure


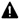
 **CRITICAL STEP:** To ensure high quality and reproducibility, all steps during RNA isolation and library preparation must be conducted using sterile, nuclease-free materials. Work with ice-chilled tubes and reagents to prevent RNA degradation and maintain the integrity of small RNA species throughout the process.

### 3.1. Isolation of Small RNAs

Cells and tissues treated with gene or cell therapy products are snap-frozen in liquid nitrogen and stored at −70 °C to preserve RNA integrity and prevent degradation until further processing.

**ADDITIONAL STEP:** Certain tissues, such as the pancreas, naturally contain high levels of nucleases, which can compromise RNA integrity. To mitigate this, additional measures may be required, such as treating samples with RNA preservatives like RNAlater Solution (Thermo Fisher) to stabilize RNA and prevent degradation.

Analysis of small RNAs can be efficiently performed using total RNA. For RNA isolation, methods such as TRIzol reagent (Thermo Fisher Scientific) or specialized RNA extraction kits designed to preserve small RNAs are recommended. Column-based isolation methods, which rely on the RNA’s negative charge and length, require kits specifically optimized for small RNA retention, such as the miRNeasy Kit for miRNA Purification (Qiagen). In all cases (Table 2), carefully follow the manufacturer’s protocol to ensure optimal RNA yield and quality.


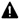
 **CRITICAL STEP:** Quality control analysis of the extracted RNA can be performed using a bioanalyzer (Agilent) according to the manufacturer’s protocol. A minimum RNA Integrity Number (RIN) of 8 or higher is expected, indicating high RNA quality. This step also allows for the detection and evaluation of the small RNA fraction present in the sample, ensuring the sample is suitable for downstream applications.


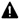
 **CRITICAL STEP:** Although small RNAs are generally well preserved, degradation of larger RNA molecules caused by repeated freeze–thaw cycles or nuclease contamination can compromise the overall quality of the library. Ensuring proper handling and storage is critical to maintaining RNA integrity and achieving reliable results.

For more detailed studies, small RNAs bound to Argonaute (AGO) proteins can be isolated through immunoprecipitation using anti-AGO2 antibodies, such as 4G8 (Wako, 015-22031) (see reference [23]). Alternatively, functional small RNAs can be isolated using the TraPR Small RNA Isolation Kit (Lexogen, Vienna, Austria), which leverages the retention of AGO complexes on a column for efficient and specific small RNA purification.


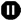
 **PAUSE STEP:** After isolation, the RNA can be stored at −70 °C for extended periods to ensure its stability and integrity.

**Table 2:** Methods of isolation of small RNAs.

Source	Advantages	Disadvantages
Total RNA (TRIzol)	High yield of RNA	Potential carryover contaminants
Total RNA (Spin Column)	High yield of RNA	Potential sequence biases *
	Reduced carryover of contaminants	
AGO immunoprecipitation	Directly addresses functional AGO-bound small RNAs	Low yield of RNA
		Increased PCR duplicates
TraPR (Separation Column)	Retention of AGO-RISC complexes	Retention of other RNA-
	Easy to use	binding protein complexes

* It has been reported negative selection of low-GC small RNAs on reduced starting material [24].

### 3.2. Preparation of Small RNA Libraries


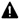
 **CRITICAL STEP:** There are multiple commercial kits and customized protocols for the preparation of small RNA libraries for NGS. Recent studies have benchmarked biases and strengths of each method (see [21,25]). In addition, many commercial providers and genomic core facilities provide small RNA sequencing as standard service.

Here, we provide a detailed description of the protocol utilized in our previous studies as an example [26]. Based on our experience with various commercial kits, these kits, although associated with a higher cost per individual library, consistently demonstrate superior efficiency in cloning small RNAs and offer greater robustness and reproducibility.

#### 3.2.1. Ligation of 3′ Adapter

1. Mix 1 μL of 5 μM RNA 3′ Adapter (RA3) with 5 μg of total RNA (starting material can range between 5 ng and 1 μg) in a final volume of 7 μL of nuclease-free water.
2. Mix the solution well and incubate the tube at 70 °C for 2 min, and then immediately place the tube on ice.
3. Prepare a mix containing:
  1 μL of 10× T4 RNA ligation buffer,
  1 μL of RNase Inhibitor,
  1 μL of T4 RNA Ligase 2 Deletion Mutant,
  In a final volume of 3 μL per reaction.
4. Mix the solution well and add 3 μL of the previous mix to the reaction tube containing the RNA and the RA3. Mix the solution by gently pipetting up and down multiple times. The final volume of each reaction is 10 μL.
5. Incubate the tube at 28 °C on a preheated block for 1 h.

**OPTIONAL STEP:** This step can be extended to an overnight incubation.

6. Add 1 μL Stop Solution (STP, 15 μM). Mix the solution by gently pipetting up and down multiple times.
7. Incubate the tube at 28 °C for 15 minutes and then place the tube on ice.

#### 3.2.2. Ligation of 5′ Adapter

1. Prepare a mix containing:
  1 μL of 7.5 μM RNA 5′ Adapter (RA5),
  1 μL of 10 mM ATP,
  0.6 μL of T4 RNA Ligase 1 (30 U/μL),
  0.4 μL of 10× T4 RNA ligase buffer,
  In a final volume of 3 μL per reaction.
2. Mix the solution well and add 3 μL of the previous mix to the reaction tube containing the small RNA–RA3 ligate. Mix the solution by gently pipetting up and down multiple times. The final volume of each reaction is 14 μL.
3. Incubate the tube at 28 °C for 15 minutes and then place the tube on ice.

#### 3.2.3. Reverse Transcription

1. Mix 6 μL of RA5–small RNA–RA3 ligate with 1 μL of RT Primer (10 μM). Mix the solution by gently pipetting up and down multiple times.
2. Incubate the reaction on a block pre-heated at 70 °C for 2 minutes and then place the tube on ice.
3. Prepare a mix containing 2 μL of 5× First Strand Buffer, 0.5 μL of 12.5 mM dNTP mix, 1 μL of 100 mM DTT, 1 μL of RNase Inhibitor, and 1 μL SuperScript II Reverse Transcriptase in a final volume of 5.5 μL per reaction.
4. Mix the solution well and add 5.5 μL of the previous mix to the reaction tube with the RA5–small RNA–RA3 and RT Primer. Mix the solution by gently pipetting up and down multiple times. The final volume of each reaction is 12.5 μL.
5. Incubate the tube at 50 °C for 1 h, and then place the tube on ice.

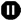
 **PAUSE STEP:** After stopping the reverse transcription, the library cDNA can be stored at −20 °C.

#### 3.2.4. PCR Amplification and Sample Barcoding

1. Prepare a mix containing:
  21 μL of ultrapure water,
  10 μL of 5× Phusion HF buffer,
  1 μL of 10 mM dNTP,
  2.5 μL of PCR5 primer,
  2.5 μL of PCR3 primer,
  0.5 μL Phusion DNA polymerase,
  12.5 μL library cDNA template.
  The final volume of each reaction is 50 μL.

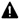
 **CRITICAL STEP:** Note that each sample will have a different PCR3 primer that contains the Illumina index to facilitate the posterior sample pooling and demultiplexing.

2. Run the PCR amplification on a thermal cycler, with the following steps:
   (Step I) 98 °C for 30 s,
   (Step II) 15 cycles of  98 °C for 10 s,
                                       60 °C for 30 s,
                                       72 °C for 15 s,
   (Step III) 72 °C for 10 min,
   (Step IV) hold at 4 °C.

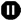
 **PAUSE STEP:** After the PCR amplification, the barcoded libraries can be stored at 4 °C for the next few days.

#### 3.2.5. Gel Purification

This step allows for enriching the library with PCR amplicons that contain small RNAs by depleting smaller amplicons that are generated during the preparation.

1. Prepare a 6% native PAGE gels by mixing:
  45 mL 6% gel,
  1 mL 10% APS,
  20 μL TEMED.
2. Pre-warm the gel by running it at 10 wats for 30 min.
3. Mix 50 μL of PCR-amplified libraries with 6 μL 10× blue juice.
4. Every few wells of the PAGE gel, load 5 μL of 20 bp DNA ladder to allow the alignment of the PCR bands to the corresponding markers at both sides.
5. Carefully load the PCR-amplified libraries with blue juice to each well.
6. Run the gel at 5 wats for at least 90 min. Allow enough separation of the different dyes on the gel.
7. Stain the gel for 3 min with Sybr Gold DNA dye diluted 1:10,000 in 1× TBE.
8. Visualize the gel on a blue-light-safe imager.
9. Identify the band between 160 and 180 bp corresponding to the size of the mature shRNA ligated with the adapters and PCR barcodes. Amplicons containing no small RNAs, shRNA products, or miRNAs are expected to appear with a size of 144 bp.
10. Extract the band by carefully cutting the window between 160 and 180 bp by aligning a clean blade with ladder markers.
11. Place the excised bands in gel breaker inserts on 2 mL tubes.
12. Centrifuge the bands placed on the 2 mL tubes with the gel breakers at 20,000× *g* in a benchtop centrifuge for 2 min.
13. Discard the gel breaker and add 300 μL ultrapure water to the gel debris.
14. Elute the library from the gel by rotating the tubes for at least 2 h at room temperature.
  Add a volume of 1000 μL to each tube with the eluted library.

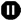
 **PAUSE STEP:** The elution can be extended to overnight at 4 °C.

15. Transfer the solution and gel debris into an inset containing a 5 μm filter.
16. Centrifuge the filter for 20 s at 600× *g* and discard the insert with the debris.
17. Prepare a mix containing:
  3 μL Glycoblue,
  30 μL 3M NaOAc,
  975 μL of pre-chilled 100% ethanol (−20 °C).

Add a volume of 1000 μL to each tube with the eluted library.

18. Incubate at −80 °C for 20–30 min to facilitate the subsequent precipitation.
19. Centrifuge at 20,000× *g* for 20 min on a benchtop centrifuge pre-cooled to 4 °C.
20. Identify the blue pellet and carefully remove the supernatant by aspirating with a 1 mL pipette from the top of the solution meniscus.

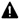
 **CRITICAL STEP:** If the pellet detaches from the bottom of the tube, spin it again at 20,000× *g* for 2 min.

21. Wash the blue pellet with 500 μL of 70% ethanol at room temperature.
22. Centrifuge at 20,000× *g* at room temperature for 2 min.
23. Identify the blue pellet and carefully remove the supernatant by aspirating with a 1 mL pipette from the top of the solution meniscus.
24. Spin the tube and remove the remaining solution with a 10 μL pipette.
25. Dry the pellet by placing the tube on a 37 °C heat block with open lid for 5–10 min (or until dry).
26. Resuspend the pellet in 10 μL of 10 mM Tris-HCI, pH 8.5, supplemented with 0.1% Tween 20.

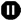
 **PAUSE STEP:** The suspended libraries can be stored at 4 °C for a day or −20 °C for longer periods.

#### 3.2.6. Library Quality Control and Sequencing

Library quality control (QC) is an important step before sequencing to ensure the integrity, size distribution, and concentration of small RNA libraries. The size of the final library is typically assessed using an Agilent Bioanalyzer—High Sensitivity DNA Chip (alternatively, TapeStation), where a peak in the ~170 bp range indicates successful small RNA library preparation with minimal adapter–dimer contamination.

Library concentration is determined using qPCR-based quantification (e.g., KAPA Library Quantification Kit) or fluorometric assays (e.g., Qubit) to enable accurate molarity estimation before pooling.


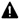
 **CRITICAL STEP:** Inaccurate library concentration measurements can lead to uneven sequencing coverage, where some samples receive excessive reads, exceeding the required depth for quantification, while others suffer from insufficient coverage, potentially compromising data quality and downstream analyses.

Indexed libraries are then pooled in equimolar amounts and prepared for sequencing on an Illumina platform (e.g., NovaSeq, NextSeq, or MiSeq) using single-end (e.g., SE75) sequencing formats. Recommended sequencing depth varies by study design, with 5–20 million reads per sample typically sufficient for characterization of overexpressed shRNAs and miRNA profiling.


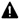
 **CRITICAL STEP:** Before loading the pooled libraries into the Illumina sequencing cartridge, denaturation with NaOH is required to ensure proper single-stranded DNA formation for sequencing. It is essential to perform this step using freshly prepared NaOH working solutions to maintain reaction efficiency and prevent degradation of the libraries.

### 3.3. Analysis of shRNA Processing and Endogenous miRNAs on Small RNA Datasets

In the following steps, we outline the data processing workflow for FASTQ files generated from small RNA sequencing. To enhance reproducibility, scalability, and user accessibility, these analyses can be conducted on cloud-based platforms such as the Cancer Genome Cloud, Cavatica, BioData Catalyst, or SPARK. Alternatively, all the referenced tools are open-source and can be installed on local computers or high-performance computing (HPC) clusters, allowing flexibility in data processing based on available computational resources.

#### 3.3.1. Adapter Removal with Cutadapt

**Figure 3:**
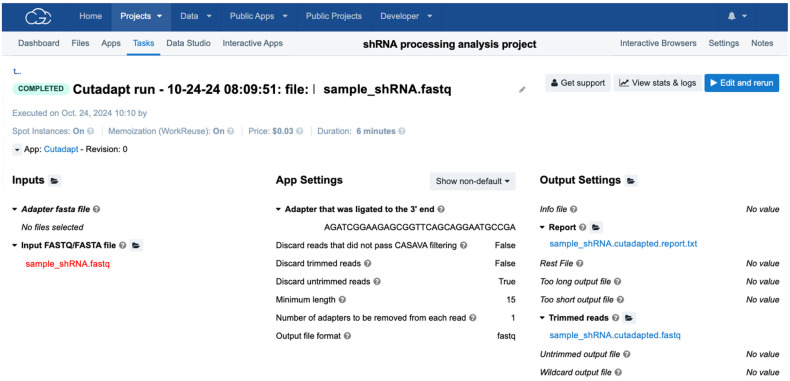
Preparation of a Cutadapt run for small RNA analysis. Steps include (**Step 1**) selecting the input sequencing files in FASTQ format, (**Step 2**) defining adapter sequences to be trimmed based on library preparation protocols, (**Step 3**) setting parameters such as minimum read length (15 nucleotides), and (**Step 4**) specifying that only trimmed treads are included in the resulting FASTQ file (cutadapted.fastq). The run also generates an informative report (cutadapted.report.txt).

1. Upload the FASTQ files to a project in the cloud computing platform.
2. Copy Cutadapt to the project.
3. Add the adapters used to generate the library in the corresponding boxes (Figure 3).
4. Select discard reads where the adapter is not found.
5. Select retain reads where the adapter is found.
6. Select to retain only reads that have a minimum length of 15 nucleotides after the adapter removal. Shorter reads are challenging to map and may result from artifacts introduced during the library cloning process. Biologically, AGO-bound small RNAs are typically at least 20–25 nucleotides in length, with shorter sequences being rare and often indicative of degradation [27,28,29].

**OPTIONAL STEP:** If necessary, the adapter removal can be performed on multiple files simultaneously by selecting the option, “Run in batch” and “Batch by file”.

For a FASTQ file of 2 GB, the adapter removal step will take around 15 min.

7. Inspect the report files. It is expected that on a FASTQ file, 75% of the reads will contain one adapter, and 74% will also fulfill the other filtering criteria previously defined.

#### 3.3.2. Interpretation of Cutadapt Reports

**Figure 4:**
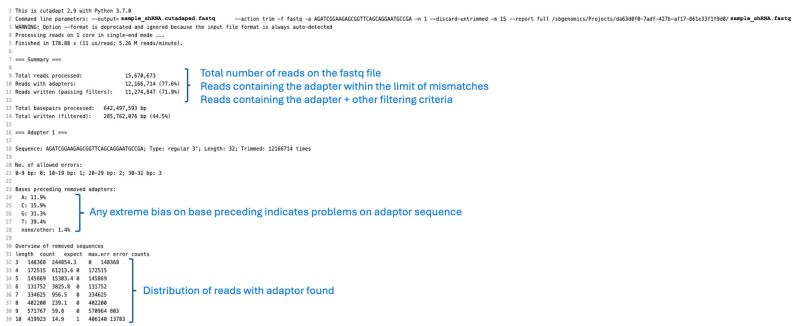
Interpretation of Cutadapt report. The figure illustrates the summary output generated by Cutadapt used for adapter trimming. Key metrics include the number of reads processed, the percentage of reads with detected adapters, and the proportion of reads successfully trimmed. The report also highlights the distribution of trimmed sequence lengths and the overall quality of the processed dataset. These results provide insights into the effectiveness of adapter removal and the readiness of the dataset for downstream analysis, such as alignment or differential expression studies.

1. Check the percentage of reads that contain a recognizable adapter and additional filtering criteria set up for the run (Figure 4).
2. Evaluate whether the distribution of bases preceding the adapter follow an unbiased distribution. Note that tailed small RNAs may overrepresent reads ending with A and T nucleotides [30,31,32].

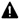
 **CRITICAL STEP:** Other biases could involve an incomplete adapter sequence.

3. Evaluate the distribution of reads and maximum number of errors allowed in each case. By default, Cutadapt allows a 0.1 error rate, thus allowing 1 error in a subsequence matching the adapter with 10 nucleotides.

#### 3.3.3. Mapping and Analysis of Small RNAs with QuagmiR

QuagmiR is a bioinformatics tool designed to analyze small RNA processing heterogeneity, including trimming, miscleavage, and tailing events, which can impact shRNA efficacy and specificity. In the original work [20], we benchmarked its performance against established isomiR mappers, including STAR [33], MicroRazerS [34], RazerS3 [35], miraligner [36], and sRNAbench [37]. The source code for QuagmiR is available for download on GitHub (https://github.com/Bofill-De-Ros-Lab/QuagmiR/ accessed on 1 December 2024) and can also be imported as a tool from the Applications Library in the Cancer Genome Cloud and other cloud-based platforms.

1. Edit the motif list file (e.g., motif_list_mmu_mirbase22.fa) with a plain text editor to include the guide and passenger strand of your shRNA.
2. In the first line of the file add a “>descriptive name” followed by a space and a unique 13-mer motif contained in the middle of all the small RNAs deriving that shRNA arm. In the second line, we provide our intended mature sequence for that shRNA (21–22 nucleotides). For example:
                            >shRNA-guide1 **GATACAGATACAT**
                            TCAG**GATACAGATACAT**AACTT
3. Repeat the same steps to include also a unique motif and the reference sequence for the passenger strand of your shRNA of interest.
                            >shRNA-passenger1 **GTAGTAGGTTGTA**
                            TGAG**GTAGTAGGTTGTA**TAGAA

4. Save the motif file, edited to contain the guide and passenger strands, along with all the other miRNA sequences expressed endogenously by the treated cells.

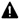
 **CRITICAL STEP:** Notice that the miRNA sequences of different organisms can differ. To this end, the QuagmiR repository contains reference files for a large list of organisms including humans, mice, rats, zebrafish, and other model organisms. https://github.com/Bofill-De-Ros-Lab/QuagmiR/tree/master/Motifs (accessed on 1 December 2024).

**Figure 5:**
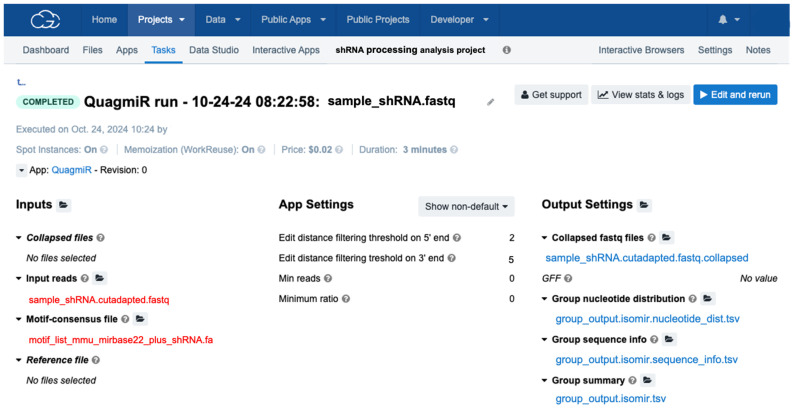
QuagmiR run for small RNA analysis. Steps include (**Step 1**) selecting the input sequencing files in FASTQ format (after adapter removal), (**Step 2**) selecting modified motif file, (**Step 3**) setting parameters such as minimum reads, minimum ratio, and number of mismatches allowed (edit distances). The run generates informative reports on overall abundances of small RNAs, isoforms, and nucleotide polymorphisms.

5. Upload the edited motif file to the project folder in the cloud computing platform.
6. Copy QuagmiR to the project.
7. Select the input cutadapted FASTQ files and motif file (Figure 5).
8. Select the number of mismatches allowed on the 5′ and 3′ end segments by defining the edit distances (Levenshtein distance). Since modifications on the 3′ end of small reads are more prevalent [27] than 5′ isoforms [26], edit distances of 5 (edit distance 3′ end) and 2 (edit distance 5′ end) are recommended.

#### 3.3.4. Interpretation of QuagmiR Reports

**Figure 6:**
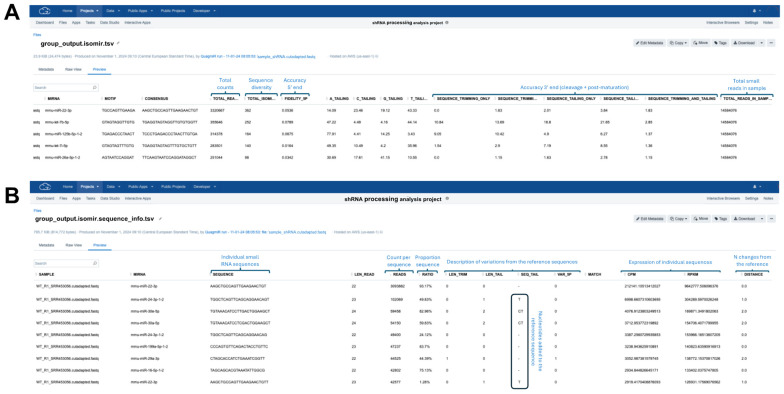
Interpretation of QuagmiR reports. (**A**) Overall abundances of small RNAs (isomir.tsv) are accompanied by overall summaries of the sequence trimming and tailing based on the reference sequence provided in the motif file. (**B**) Detailed report at the isoform level (isomir.sequence.tsv) includes sequence abundance and descriptors of 5′ and 3′ modifications.

1. Examine the relative abundance of the guide and passenger strands of the shRNA in the summary report (Figure 6A). In applications involving transient shRNA expression, both strands may be among the most highly expressed small RNAs, which is a common observation. Monitoring their relative proportions provides insights into strand selection efficiency and potential off-target effects.
2. Evaluate 5′ end processing accuracy using the Fidelity_5P metric in the summary report (Figure 6A). This metric quantifies the heterogeneity of 5′ isoforms as a weighted average, typically reflecting variability in cleavage site selection by DROSHA or DICER1. Values range from 0 for highly precise processing to 1 or 2 for inaccurately processed shRNA scaffolds, where increased heterogeneity suggests suboptimal cleavage efficiency.
3. Examine 3′ end heterogeneity in the summary report (Figure 6A), which is assessed through multiple parameters, including the number of isoforms as well as the percentage of sequence trimming and tailing. Unusually high values in any of these metrics may indicate suboptimal shRNA processing, such as premature termination or imprecise cleavage, or post-maturation modifications, such as target-directed microRNA degradation (TDMD).
4. For a more detailed analysis of the generated isoforms, inspection of sequence-level reports is recommended (Figure 6B). This allows for the sorting of reads and the identification of isoform sequence compositions, particularly those with altered 5′ ends. Additionally, this analysis can help determine whether the presence of 3′ isoforms results from shRNA scaffold misprocessing (templated isoforms) or from endogenous cellular processing mechanisms (non-templated isoforms).

### 3.4. Calculation of Advanced shRNA Biogenesis Metrics

To comprehensively assess shRNA processing efficiency and accuracy, advanced biogenesis metrics are calculated either in Excel or using R scripts. These metrics quantify key aspects of strand selection and cleavage precision, providing insights into the molecular processing of shRNAs by DROSHA, DICER1, and Argonaute proteins.

#### 3.4.1. Analysis of shRNA Strand Selection

Optimal strand selection is important for effective gene silencing and relies on the incorporation of the guide strand into the RNA-induced silencing complex (RISC). Any reads corresponding to the passenger strand indicate potential off-target effects, as they may be inadvertently loaded into Argonaute proteins. Additionally, preferential incorporation of the passenger strand can reduce the availability of the guide strand, thereby compromising the silencing efficiency of the target RNA.

The strand selection index can be calculated as follows:


Strand selection index = CPM guide/(CPM guide + CPM passenger)
(1)

Note that an efficient shRNA design will yield a strand selection index of 0.9 or higher.


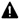
 **TROUBLESHOOTING:** Suboptimal strand selection can occur if the 5′ nucleotide of the guide strand is not optimal for Argonaute loading. Structural studies have demonstrated that Argonaute proteins preferentially incorporate guide strands that begin with a uridine (U) or adenine (A) at the 5′ end [38], as well as thermodynamic features of the shRNA duplex [39].

#### 3.4.2. Analysis of shRNA 5′ End Isoforms

5′ End Variability and Its Impact on shRNA Processing

Another key metric assesses variability at the 5′ end of the guide strand, which arises from imprecise DROSHA and DICER1 cleavage during shRNA processing. Variability at this position is often indicative of inefficient substrate recognition and binding by either endonuclease. Moreover, 5′ end heterogeneity can disrupt Argonaute (AGO) loading, leading to arm switching, where the unintended strand is preferentially incorporated into AGO, thereby reducing the effectiveness of the intended guide strand. Additionally, variability in the 5′ seed sequence can result in “seed shifting”, where the guide strand gains off-target interactions through miRNA-like effects, expanding its unintended target repertoire.

To quantify these effects, we adapted the effective number (inverse Simpson index) equation from Laakso and Taagepera (1979), where p represents the proportion of reads containing a specific seed sequence. This metric provides a robust measure of cleavage fidelity and its impact on shRNA specificity.

The effective number of “seeds”, or the N seed index, can be calculated as follows:

 N effective seed = 1/(p^2^_seed_1_ + p^2^_seed_2_ + … + p^2^_seed_n_) (2)

Note that an efficient shRNA design will yield an N seed index between 1 and 1.1.


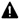
 **TROUBLESHOOTING:** Suboptimal generation of guide sequences can indicate defects in the shRNA scaffold. The use of reported scaffolds such as pri-miR-22 or pri-miR-16 is encouraged. To ensure proper processing, it is also a good practice to evaluate the folding of the shRNA scaffold with RNAfold [40], Mfold [41], or similar RNA-folding algorithms.

## 4. Expected Results

**Figure 7:**
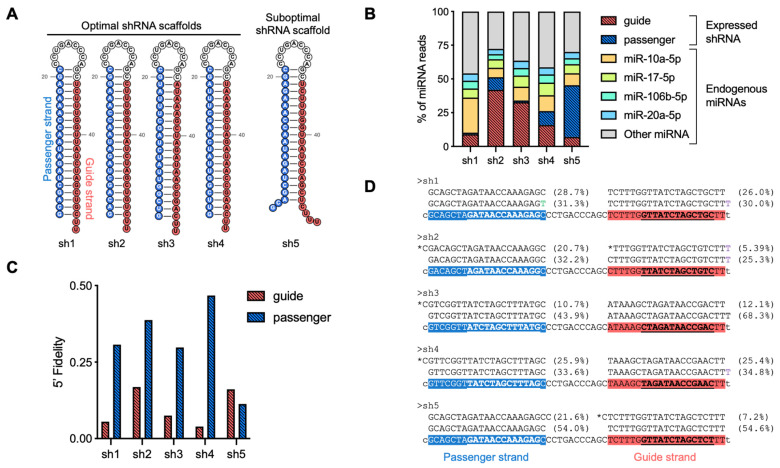
Analysis of shRNA biogenesis. (**A**) Schematic representation of the shRNA structures analyzed in this study. (**B**) Quantification of the proportion of mapped reads corresponding to shRNA-derived products and endogenous miRNAs. (**C**) Evaluation of 5′ end fidelity for both the guide and passenger strands, reflecting processing accuracy. (**D**) Representative examples of the most abundant small RNA-seq reads observed for each shRNA construct. Highlighted: non-templated nucleotides (green) and ambiguous nucleotides (purple); isoforms with different seed (asterisk), passenger strand (blue), and guide strand (red); motifs used for QuagmiR analysis (bold and underlined).

To demonstrate the analysis of shRNAs outlined in this protocol, we present an example featuring five shRNAs expressed under the control of the U6 Pol III promoter. In each construct, transcription was designed to initiate at the G nucleotide at position 1, followed by the passenger strand, a terminal loop, and the guide strand, with transcription termination occurring at a poly-U track (Figure 7A). For sh1 to sh4, the resulting hairpins were designed to include the characteristic 2-nucleotide 3′ overhangs, optimizing them for efficient DICER1 processing. In contrast, sh5 was intentionally structured to form a suboptimal hairpin, potentially affecting its processing efficiency.

Each construct was individually transfected into HEK293 cells, and total RNA was extracted 72 h post-transfection for small RNA sequencing analysis. Libraries were prepared, and the resulting FASTQ files were processed using the described pipeline, incorporating Cutadapt for adapter trimming and QuagmiR for alignment and quantification. The relative abundance of each shRNA-derived product was then analyzed within the pool of endogenously expressed microRNAs in the treated cells. The results show that shRNA-derived reads, including both guide and passenger strands, account for 10% to 45% of the total mapped reads (Figure 7B). Additionally, sh1 and sh3 exhibit a strong preference for guide strand retention, whereas sh2 and sh4 show substantial incorporation of the passenger strand into Argonaute proteins. Notably, sh5 demonstrates a skewed strand selection, favoring the passenger strand over the intended guide strand, suggesting inefficient strand bias or suboptimal processing.

Next, we analyzed the 5′ end homogeneity of the guide and passenger strands (Figure 7C). The data show that guide strands (located on the 3p arm) generally undergo more precise processing, whereas passenger strands (on the 5p arm) exhibit greater variability. This aligns with the precise processing of 3p arms by DICER1 [10], whereas 5p arm variants may be affected by transcription start site variability linked to the U6 Pol III promoter [42]. Among the constructs, sh2 and sh5 show the poorest processing accuracy. In the case of sh2, this reduced precision may result from the absence of an adenine (A) or uridine (U) at the 5′ end of the guide strand, a feature known to enhance Argonaute loading efficiency [38]. For sh5, the lack of a canonical 3′ overhang for DICER1 recognition and binding may contribute to an increase in miscleavage events, leading to greater processing variability [15]. However, identifying specific structural defects would require detailed mechanistic studies.

Finally, we present the dominant reads identified in the small RNA-seq data for each construct (Figure 7D). This manual inspection provides a detailed view of the major products generated during shRNA biogenesis, highlighting their processing efficiency and interactions with the endogenous RNA metabolism. Additionally, this analysis reveals the presence of tailed and trimmed isoforms, which likely result from post-transcriptional modifications and RNA decay pathways.

## 5. Conclusions

While shRNA technology is a powerful tool for gene silencing, several limitations must be considered to ensure its effective application (Table 3). Off-target effects, often caused by improper DROSHA and DICER1 processing, can generate unintended RNA fragments that enter the RNA-induced silencing complex (RISC) and regulate non-target transcripts through miRNA-like repression. Additionally, suboptimal shRNA design, including sequence selection and structural inefficiencies, can result in passenger strand incorporation into AGO, further expanding the off-target repertoire. Variability in transcription start sites, alternative cleavage events, and improper guide strand selection may also compromise specificity. Moreover, oversaturation of the endogenous RNAi machinery can interfere with the normal function of endogenous miRNAs, leading to unintended cellular consequences. Addressing these limitations requires rigorous computational prediction, transcriptome-wide off-target screening, and biochemical validation to optimize shRNA design and processing for improved specificity and reduced off-target effects. A critical evaluation of these factors is essential to advancing the safe and effective therapeutic application of shRNA technology.

## Figures and Tables

**Table 1:** RNAi designs in gene and cell therapy.

Source	Advantages	Disadvantages
Synthesized siRNA	Can be chemically modified for enhanced stability and reduced immunogenicity	Effects are transient
	No biogenesis steps involved	Delivery challenges
Pol-III-driven shRNA (e.g., U6 or H1)	Can be encoded on a viral vector	Increased cellular toxicity
	High intracellular expression	Unintended off-targets
	Long-term silencing in stable cells	Limited number of promoters
Pol-II-driven pri-shRNA (e.g., CMV, Tet-ON/OFF)	Can be encoded on a viral vector	Lower intracellular expression
	Lower risk of off-targets	Higher biogenesis complexity
	Multiple promoter options	

**Table 3:** Best practices to enhance shRNA specificity and minimize off-target effects.

Best Practice	Rationale
Guide Selection	
Target Site Selection	Use bioinformatics tools to predict and minimize off-target effects by selecting sequences with high specificity for the target mRNA [43,44].
Seed Region Optimization	Avoid pairing in the seed region (nucleotides 2–8) to unintended transcripts or highly prevalent k-mers in the 3′UTR [45].
**Hairpin Design**	
Optimized Hairpins	Design shRNAs with loop structures that promote efficient DROSHA and DICER1 processing, reducing heterogeneous processing and off-target effects [3].
Strand Selection	Ensure preferential loading of the intended guide strand into RISC by modifying thermodynamic asymmetry or using mismatches in the passenger strand [46].
**Evaluate Off-Targets**	
Off-Target Screening	Use CLIP-based approaches (AGO-CLEAR CLIP, PAR-CLIP) to identify unintended targets transcriptome-wide [47].
Mismatched Controls	Use control shRNAs with single or double mismatches to distinguish specific from off-target effects [48].

## Data Availability

Figures in this protocol were generated from the reanalysis of GSE108893 dataset deposited in NCBI GEO Datasets.
